# Recent advances in the genetics of preterm birth

**DOI:** 10.1111/ahg.12373

**Published:** 2019-12-19

**Authors:** Megan Wadon, Neena Modi, Hilary S. Wong, Anita Thapar, Michael C. O'Donovan

**Affiliations:** ^1^ MRC Centre for Neuropsychiatric Genetics and Genomics Institute of Psychological Medicine and Clinical Neurosciences, Cardiff University School of Medicine Cardiff Wales; ^2^ Section of Neonatal Medicine, Department of Medicine Chelsea and Westminster Hospital Campus, Imperial College London United Kingdom; ^3^ Department of Paediatrics University of Cambridge Cambridge United Kingdom

**Keywords:** genetics, gestational age, preterm birth

## Abstract

Preterm birth is associated with short‐ and long‐term impairments affecting physical, cognitive, and neuropsychiatric health. These sequelae, together with a rising preterm birth rate and increased survival, make prematurity a growing public health issue because of the increased number of individuals with impaired health throughout the life span. Although a major contribution to preterm birth comes from environmental factors, it is also modestly heritable. Little is known about the architecture of this genetic contribution. Studies of common and of rare genetic variation have had limited power, but recent findings implicate variation in both the maternal and fetal genome. There is some evidence risk alleles in mothers may be enriched for processes related to immunity and inflammation, and in the preterm infant, processes related to brain development. Overall genomic discoveries for preterm birth lag behind progress for many other multifactorial diseases and traits. Investigations focusing on gene–environment interactions may also provide insights, but these studies still have a number of limitations. Adequately sized genetic studies of preterm birth are a priority for the future especially given the breadth of its negative health impacts across the life span and the current interest in newborn genome sequencing.

## 1 INTRODUCTION

Birth is defined as preterm if it occurs before 37 weeks of gestation, and accounts for ∼60,000 births in the United Kingdom every year (Blencowe et al., 2012). It is further categorized as very preterm (birth before 32 weeks gestation) and extreme preterm (birth before 28 weeks gestation). Preterm birth can also be categorized into medically indicated and spontaneous. The former is where labor is induced or a Caesarean section is performed because of medical risks or complications, the latter where onset of labor occurs spontaneously prior to 37 weeks gestation. However, this distinction fails to take into consideration the possibility that medical conditions necessitating delivery by medical intervention has factors in common with “spontaneous” preterm birth.

Globally, in 2010, preterm births were estimated to account for ∼11.1% of all live births and evidence suggests this rate is increasing (Blencowe et al., 2012). The survival of very preterm infants has improved steadily over recent years and is now over 90% in high‐income settings (Blencowe et al., 2012; Santhakumaran et al., 2018). However, globally, complications related to preterm birth remain a major cause of neonatal and under‐5 mortality (Liu et al., 2015). Preterm individuals also are at substantially higher risk of impairments affecting multiple organ systems (Haraldsdottir et al., 2018; Huang, Lin, Wang, Su, & Lin, 2018; Parkinson, Hyde, Gale, Santhakumaran, & Modi, 2013), including potentially disabling neurological and neuropsychiatric disorders (Agrawal, Rao, Bulsara, & Patole, 2018; Franz et al., 2018; Heuvelman et al., 2018; Nosarti et al., 2012; Sutton & Darmstadt, 2013). This highlights the importance of determining the mechanisms behind preterm birth and how these mechanisms might relate to the difficulties faced later in life. Although environmental factors are known to contribute to preterm birth risk, there is growing evidence that highlight the contribution of genetic factors. In this article, we aim to provide a brief review of genetic findings for preterm birth and highlight issues that need to be addressed in future research.

## 2 TWIN AND FAMILY STUDIES

Preterm birth runs in families. The risk of preterm birth for women who have a mother or a sister with a history of having delivered preterm is higher than that observed in the general population (Sherf et al., 2017). There have been a number of twin studies, all of which find that there is a significant, genetic contribution to preterm birth. As is typical of this type of study, observed heritability estimates have varied (Table 1), ranging from approximately 15% (Lunde, Melve, Gjessing, Skjaerven, & Irgens, 2007; Wu et al., 2015) up to 30%–40% (Clausson, Lichtenstein, & Cnattingius, 2000; Kistka et al., 2008; Plunkett et al., 2009) for the maternal genetic contribution. These estimates should be interpreted with caution, as they may not account for potential confounding; for example, an observed maternal genetic effect may also include the effects of the fetal genome (York, Eaves, Neale, & Strauss, 2014) as well as lifestyle factors that are socially transmitted from mothers to daughters (Svensson et al., 2009). A large Swedish registry‐based (*n* = 244,00) extended twin‐sibling design study showed that fetal genetic factors contributed 13.1% of the genetic variation to gestational age at delivery (York et al., 2013). This study was important in also showing that the largest contribution came from environmental factors, specifically pregnancy‐specific factors (56%). However, another large study of 630,000 families observed minimal fetal contribution, while maternal genetic factors explained 25% of the variation in preterm birth (Svensson et al., 2009). Paternal effects have been less commonly investigated, with either negligible effects (Kistka et al., 2008) or a small paternal contribution (6%) (Wu et al., 2015) being found. Consistent with a paternal contribution, it has been reported that maternally reported ancestry, including paternal ancestry, modifies risk for preterm birth (Palomar, DeFranco, Lee, Allsworth, & Muglia, 2007).

**Table 1:** Twin studies of gestational age and preterm birth

Heritability estimate	Authors	Study type	Variable measured
36% (maternal)	Clausson et al., 2000	Twin study investigating concordance and correlation between twin mothers of differing zygosity (potential confounding from fetal genome)	Gestational age (no distinction between spontaneous and medically indicated onset of labor)
14% (maternal) 11% (fetal)	Lunde et al., 2007	Parent–offspring study using path analysis and maximum likelihood principles and Pearson's correlation coefficients for different familial relationships	Gestational age (births by Caesarean section were excluded as these births are more likely to be medically indicated)
34% (maternal) Negligible (paternal)	Kistka et al., 2008	Extended twin design investigating the correlations between monozygotic twins and first‐degree relatives reporting on the gestational age of their first‐born child (potential confounding from fetal genome)	Gestational age (no distinction between spontaneous or medically indicated onset of labor)
44% (maternal) 33% (fetal)	Plunkett et al., 2009	Family study with mother–infant pairs using segregation analysis and estimating the parameters using maximum likelihood method (potential confounding between maternal and fetal estimates)	Gestational age (spontaneous onset of labor)
27% (maternal)	Treloar et al., 2000	Twin study (potential confounding from fetal genome)	Any preterm birth (no distinction between spontaneous or medically indicated onset of labor)
20.6% (maternal) 13.1% (fetal)	York et al., 2013	Extended twin‐sibling design investigating the offspring of twins, full siblings, and half‐siblings using structural equation modeling. (excluded multifetal pregnancies)	Gestational age (exclusions for some medical reasons including preeclampsia and placental abruption)
25% (maternal) (29% for spontaneous; 13% for indicated) 5% (fetal) (negligible for spontaneous; 14% for indicated)	Svensson et al., 2009	Family study investigating children of siblings using an alternating logistic regression model	Preterm birth (live birth at <37 weeks gestation) (data were analyzed as a combined group including spontaneous and medically indicated onset of labor and also as two separate groups)
14.21% (maternal) 6.77% (paternal) 13.33% (narrow‐sense) 24.45% (broad‐sense)	Wu et al., 2015	Family study assessing the correlation of gestational age between offspring and full/half siblings and a regression analysis in parent offspring pairs; narrow‐sense heritability was calculated as 4 times the paternal half‐sib correlation and broad‐sense heritability was calculated as the narrow sense heritability plus the full sibling correlation (representing the dominance variance) (not able to control for environmental risk factors)	Gestational age (excluded births that were unlikely to have a spontaneous onset of labor)

One problem is that studies have used inconsistent definitions of preterm birth. For example, Lunde et al. (2007) (Table 1) excluded births before 35 weeks gestation and investigated gestational age as a quantitative trait, and others have used a categorical variable of preterm birth (<37 weeks) (Treloar, Macones, Mitchell, & Martin, 2000). Also, some studies have not differentiated between spontaneous and medically indicated preterm birth, and others have focused on spontaneous preterm birth. This may be relevant as in the large study of Svensson et al. (2009), for the medically indicated preterm birth group, the maternal genetic contribution was half that (13%) of the spontaneous group (27%). The fetal genetic contribution was 14% in the medically indicated group compared with none in the spontaneous group, although it should be noted the confidence around this estimate of 14% included 0%. Overall, despite the differences in studies, findings suggest estimates of heritability that are modest for the maternal genome, and considerably weaker, possibly negligable, from the fetal genome.

## 3 GENOME‐WIDE ASSOCIATION STUDIES (GWAS): SEARCHING FOR COMMON GENE VARIANTS

### 3.1 Maternal GWAS

Effect sizes are expected to be particularly weak for common alleles that influence liability to a phenotype, which is likely to be subject to negative selection, probable for preterm birth given its associated infant mortality and morbidity. Unsurprisingly then, given their small sample sizes, the first few GWASs based on the maternal genome (*n* = 1,881 [Zhang et al., 2015]; *n* = 3022 [Myking et al., 2013]) did not yield significant associations.

The largest maternal GWAS to date included 43,568 mothers of European ancestry of whom 3,331 reported a gestational duration <37 weeks for their first live singleton birth where onset of labor was spontaneous (Zhang et al., 2017). Promising findings were further tested in a sample of 8,643 individuals (2,565 preterm). In the combined sample, six genetic loci were significantly associated with gestational duration, of which three were associated with preterm birth. The same loci were also found in the fetal genome; however, the association effect sizes were around half that of those conferred by the maternal genotypes. This suggests the associations are driven by the maternal genome (the allele frequencies of the fetus being passively elevated as a consequence of the elevated allele frequencies in the mothers). These findings suggest that, at least in part, from the perspective of genetic liability, preterm birth represents the tail of a continuous trait of gestational age. It is the case that complex phenotypes in general behave as quantitative traits, but it cannot be taken as certain that all alleles that influence those traits are efficiently observed in general populations reflecting the full range of trait outcomes. In the neurodevelopmental field, for example, there are mutations of large effects but whose frequencies are so rare that they are essentially not observed in unselected samples of the size that it is currently feasible to study. Similar considerations may hold for very preterm births where it is possible that there may be some variants, for example, highly penetrant deleterious de novo mutations, which may have very large effects on fetal development and the age at birth, such that are also only observed in the very preterm range.

As is typical for complex phenotypes that are under negative selection, the effect sizes of the common GWAS associations are small and explain only a trivial fraction of the heritability of preterm birth. Nevertheless, the ability to find preterm‐associated alleles in what is still a relatively small (in GWAS terms) study suggests that the GWAS approach is likely to yield many more findings as sample sizes increase.

### 3.2 Fetal/offspring GWAS

The first GWAS based on the fetal genome of spontaneous preterm birth (<37 weeks gestation) failed to identify any associated loci (Myking et al., 2013). The second GWAS of the fetal genome investigated spontaneous preterm birth <34 weeks gestation ( Zhang et al., 2015); two variants achieved genome‐wide significance. However, with a sample of only 916 cases (total neonatal sample size 1,851), as well as other limitations (Table 2), the initial associations are at best highly preliminary (as recognized by the authors) and, we suggest, more likely to be spurious rather than true associations.

**Table 2:** Genome‐wide association studies of preterm birth

Authors	Category of preterm birth	Sample size	Limitation	Concerns
Zhang et al., 2015	<34 weeks gestation (spontaneous onset of labor)	1,851 (916 cases)	Small sample size	Reduces power
			Inclusion of highly heterogeneous groups (in terms of ancestry)	Further reduces power
			Lack of stringent control for background ancestry	Increases false‐positive rate
			Lack of support from markers in the same chromosomal regions	Increases probability of genotyping errors
			Failure of replication in independent samples	Suggests unreliable results
Rapport et al. 2018	Birth between 25 and 30 weeks gestation (spontaneous onset of labor)	13,944 (1,349 cases) split into five ancestral groups EUR 9,890 (260 cases) AFR 1874 (190 cases) AMR 1847 (745 cases) EAS 249 (131 cases) SAS 59 (23 cases)	Each association was only observed in one of the five samples	
			Unable to obtain replication in any external samples	Suggests unreliable results
			Samples from which the associations were seen were small (AFR *N* preterm = 190, AMR *N* preterm = 745)	Reduces power
			Each subsample was genetically heterogeneous, controlling for ancestry challenging	Further reduces power
			Cases and controls were taken from different populations	Possible genotyping batch effects

A larger GWAS investigated spontaneous preterm birth between 25 and 30 weeks gestation in a total sample size of 13,944, of whom 1,349 met the preterm criterion, split into five ancestry groups (Rappoport et al., 2018). The authors reported two significant associations. Again, these findings have to be viewed with caution for many of the reasons outlined above (Table 2), including each association only being seen in one of the five small samples (African ancestry sample *N* preterm birth = 190, American sample 745). One interpretation of the findings is that it suggests different alleles exist in people of different ancestries, and this may partly explain differences in preterm birth rates seen in different populations. However, this should also be viewed with caution. First, the individual findings in each population are not yet robust. Second, it is well known that in underpowered studies such as the subgroups here represented, even in the face of genetic homogeneity, sampling variance makes replication across studies unlikely. Nevertheless, should more secure findings of differences in the architectures across ancestral (or indeed ethnic) groups be identified, it will be important to investigate interactions with variable environmental exposures that also differ if the findings are to translate to reducing the disparity in preterm births (Burris et al., 2019).

The largest published GWAS to date included 84,689 children; 4,775 were preterm of whom 1,139 of these were considered early preterm infants (<34 weeks) (Liu et al., 2019). Infants were excluded if their birth was medically indicated. The study reported association between markers in the fetal genome at 2q13 and gestational duration, and, moreover, the allele that was associated with increased gestation was also associated with postterm birth greater than 42 weeks. Overall, 7.3% of the variance in gestation duration was explained by common fetal genetic variants. There were no replicated associations with either preterm birth or early preterm birth and, interestingly, the allele associated with gestational age was reported to show no association with preterm or early preterm birth. This suggests the genetic variants that influence the early extreme of gestational age may differ from those that influence gestational age in general, although the lack of association may also reflect the incomplete power of the study.

To summarize the GWAS findings for preterm birth, thus far studies have, in general, been underpowered. Nevertheless, they strongly suggest that common alleles influencing preterm birth with even modest (odds ratio [OR] > 1.5) effect sizes do not exist, but as shown by Zhang et al. (2017), alleles with smaller effects do exist and they can be captured by larger GWASs. It is likely that large‐scale population studies of gestational age can uncover some of the heritability attributable to common variation, although it is not yet clear whether at the extremes of preterm birth, the trait shows some qualitative as well as quantitative differences from gestational age.

Sample sizes lag behind GWASs of many complex diseases and traits, for example, increasing the sample size to include 36,989 cases and 113,075 controls when using GWAS to investigate schizophrenia resulted in the discovery of 83 novel associated loci (out of 108 associated loci found) (Schizophrenia Working Group of the Psychiatric Genomics Consortium, 2014), a pattern that also holds true for other traits (Warren et al., 2017). These common gene variants only partially explain observed twin heritability and it is also important to realize that extensive correlations between genotypes at associated loci means the index single‐nucleotide polymorphisms identified by GWAS may not necessarily be those that directly contribute to risk. However, the hope is that common gene variant discovery ultimately will provide valuable insights into biological mechanisms, and thereby identify potential novel therapeutic targets, and inform risk prediction and disease stratification. For example, there is growing interest in whether polygenic risk scores could add value to current risk‐prediction models used for disease onset prediction (e.g., coronary artery disease) and be used in combination with family history and clinical variables for predicting prognosis as well as disease onset (Lambert, Abraham, & Inouye, 2019).

Meta‐analysis of large population‐based or case‐register cohorts of hundreds of thousands (or more) will likely provide sufficient power to identify hundreds of GWAS loci associated with gestational age. However, for assuring the validity of the findings for clinically relevant phenotypes, particularly very preterm birth (as well as for identifying the contribution of rare alleles of large effect), large‐scale (tens of thousands) targeted recruitment from clinics is likely to be needed.

## 4 RARE VARIANTS

### 4.1 Copy number variants

Some investigators have studied the role of copy number variants (CNVs) in preterm birth, but few have taken a whole genome perspective, focusing instead on specific candidate CNVs. The single study we are aware of that has taken a genome‐wide approach found no specific CNV associations in the genomes of 454 mothers giving birth before 34 weeks (most of whom had spontaneous onset of labor) compared with 1,018 mothers who delivered at term, nor was there any difference in overall CNV burden between the groups (Uzun et al., 2016a).

The most intensively studied candidate region for CNVs has spanned *glutathione‐S‐transferase (GST)* genes, involved in the metabolism of toxins, including a maternal deletion CNV of approximately 28 kb at the *glutathione‐S‐transferase theta 1 (GSTT1)* in spontaneous preterm birth at <37 weeks gestation (Zheng et al., 2013). Meta‐analysis of nine studies (including 2,526 cases and 4,565 controls) found evidence for association (Liu, Tang, Chen, & Huang, 2014), but the deletion CNV at *GSTT1* is common (15.8%) and if genuinely associated, confers a weak effect size in the typical common variant range (OR ∼1.18). Another study of 231 mothers of infants born preterm and 378 control mothers (188 infants born preterm and 391 infants born at term) observed a trend for association with maternal insertion at *glutathione‐S‐transferase Mu 1 (GSTM1)* and protection against preterm birth, but the primary driver of the association appeared to be acting via the fetal genome (Bustamante et al., 2012). These results support previous findings that suggest maternal *GSTM1‐/GSTT1‐*(null) genotype increases the risk for preterm birth (Mustafa et al., 2010), but with major caveats. It is unclear whether the statistical evidence (association statistics were not reported in the meta‐analysis) meets the standard of genome‐wide significance that is now widely required in the field of human genetics, even for candidate genes, but with the reported sample and effect sizes, this seems unlikely. Further (larger) stringently conducted studies are required to definitively answer whether CNVs at these loci confer liability to preterm birth, and if so, whether this is a maternal or fetal effect.

### 4.2 Whole‐exome and ‐genome sequencing

To date, sequencing studies of preterm births have been small, typically focusing on multiplex families in which more than one woman has had a child born prematurely, the premise being whether such families might be segregating a rare allele with large effect.

#### 4.2.1 Maternal rare coding and noncoding variants

McElroy and colleagues performed exome sequencing on a small sample (10 cases including two mother–daughter pairs) to select variants to genotype in a larger sample (237 cases of spontaneous preterm birth, 328 controls) (McElroy et al., 2013). No genome‐wide significant findings emerged. Uzun et al. (2016b) in a study based on 32 women who had given birth preterm (with a family history of preterm birth) and 16 controls sequenced 329 candidate genes but again observed no significant associations with preterm birth. While the focus on candidate genes in these studies may be considered pragmatic given the sample sizes, the failure of the candidate gene approach for other complex phenotypes suggests that in most cases, the choice of candidates is not much better than random for identifying associated genes. It is therefore widely considered that regardless of the number of genes sequenced, genome‐wide significant thresholds are still appropriate.

A recent whole‐exome study was conducted in 17 mothers from seven Finnish multiplex families, 13 of whom had experienced spontaneous preterm birth, and 10 of them had experienced recurrent spontaneous preterm birth. A further 93 sister pairs and two sister triads were used as a “replication population.” A number of biological pathways were identified that were enriched for genes containing rare variants predicted to be damaging (Huusko et al., 2018). One of the main drivers of pathway enrichment was the gene *HSPA1L* (encoding *heat shock protein family A member 1 like*). The authors next examined imputed alleles at this locus in a GWAS dataset of over 40,000 mothers (approximately 3,300 cases); of two variants that were testable in that dataset, one was nominally associated (*P* = 0.0022). Although some evidence was provided that the associated allele might confer differential endometrial fibroblast responsiveness to glucocorticoids, the associations did not replicate in two other (small) datasets and use of imputed data for alleles of very low frequency is not necessarily reliable. It will be necessary for this gene to obtain much stronger support before it can be decisively considered to be involved in preterm birth.

#### 4.2.2 Fetal rare coding and noncoding variants

Modi and colleagues recently presented a series of studies utilizing exome sequencing of the fetal genome to identify rare variants in a number of candidate genes in a small (but variable) number of cases of preterm premature rupture of membranes (PPROM), a subcategory of spontaneous preterm birth, as well as a small (variable) number of normal term control mothers. They reported variants in genes involved in the structural integrity of fetal membranes (Modi et al., 2017a), innate immunity (Modi et al., 2017b), and other pathways (Modi et al., 2018) but in none of the studies was a clear relationship established between PPROM and the genetic variants observed.

The final study we consider here adopted a whole‐genome sequencing approach (Li, Oehlert, Snyder, Stevenson, & Shaw, 2017). Rather than focus on cases and controls, the authors used 816 parent‐proband trios (295 with preterm birth) to identify de novo genetic variants. While this study was underpowered to detect specifically associated genes and did not distinguish between medically indicated and spontaneous preterm birth, it did provide evidence that those born preterm have an increased rate of de novo variants at a genome‐wide level. Also when damaging de novo variants were observed in those born preterm, they were more likely to occur in genes that do not tolerate mutations well, and that are expressed in early fetal brain development (Li et al., 2017). While not definitive, this study does provide evidence that suggests further investigation of rare de novo genetic variants in preterm birth will be important.

## 5 GENE–ENVIRONMENT INTERACTION

Environmental as well as genetic factors contribute to preterm birth, and this has led some to look for gene‐environment interaction. Examples include possible relationships between candidate genes involved in metabolic detoxification and exposure to pollution (Suh et al., 2008), organochlorine pesticides (Mustafa, Banerjee, Ahmed, Tripathi, & Guleria, 2013), benzene, a chemical found in cigarette smoke (Wang et al., 2000), and maternal smoking (Tsai et al., 2008). Nominally significant reports of interactions have been reported in these studies (some distinguishing between spontaneous and indicated preterm birth and some not) but the sample sizes are too small to generate robust results. The studies have not controlled for genome‐wide testing, the underpinning candidate genes have often had larger effect sizes than seem feasible based on the larger studies discussed above, and the findings are as yet unsubstantiated by replication.

There is only one study we are aware of that has taken a genome‐wide approach to investigate gene–environment interactions in preterm birth investigating spontaneous preterm birth separately and combined with medically indicated preterm birth. This analysis (*n* = 1,733, including 698 cases) based on African‐American women reported genome‐wide significant interactions between variants in *COL24A1* and pre‐pregnancy body mass index on risk of preterm birth; this finding was subsequently replicated in 300 African‐American women (Hong et al., 2017). It is important to note that studying gene–environment interactions poses many challenges above and beyond straightforward GWAS (Hunter, 2005), including seemingly simple matters such as how to define and model interaction, practical issues such as larger sample size requirements than simple gene discovery, and design issues including the possibility of introducing confounding. As has been the case in complex disorders more widely, the potential for false‐positive findings is high and therefore even the most promising of the current findings should be viewed with caution (Boffetta et al., 2012).

## 6 CONCLUSIONS

Preterm birth is a major public health issue, with individuals at substantially higher risk of a wide range of adverse health outcomes that extend throughout the life‐course and into the next generation. It is a multifactorial outcome that is the result of multiple environmental and genetic (maternal and fetal) risk factors. Gene discovery for preterm birth is lagging behind many other multifactorial disorders, most likely because GWASs of gestational age and preterm birth have been relatively small. However, emerging evidence suggests there are a large number of alleles involved, each contributing to a small amount of population liability. Sample sizes also still need to increase to detect likely causal de novo and inherited rare genetic variants. Preliminary investigations suggest rare, deleterious genetic variants could be enriched in both mothers and offspring, although no robustly associated variants have emerged. Gene–environment studies may provide further insight, but the current findings need rigorous testing. Advancements in statistical methods, such as polygenic risk scoring may also help with prediction of preterm birth, enabling women and prospective parents to make informed lifestyle choices (Torkamani, Wineinger, & Topol, 2018). However, so far, polygenic risk scores are only weakly predictive for many diseases, and not at all predictive for preterm birth. Given the growing global burden of preterm birth, much larger genetic studies are of relevance and importance, and collaborations across different groups are likely to be important for generating adequate sample sizes. This is likely to become a pressing scientific question with growing interest and debates about newborn genome sequencing (Berg & Powell, 2015; Berg et al., 2017).

## CONFLICTS OF INTEREST

The authors state that they have no conflict of interest.
